# Development of a real-time flexible multiphoton microendoscope for label-free imaging in a live animal

**DOI:** 10.1038/srep18303

**Published:** 2015-12-17

**Authors:** Guillaume Ducourthial, Pierre Leclerc, Tigran Mansuryan, Marc Fabert, Julien Brevier, Rémi Habert, Flavie Braud, Renaud Batrin, Christine Vever-Bizet, Geneviève Bourg-Heckly, Luc Thiberville, Anne Druilhe, Alexandre Kudlinski, Frédéric Louradour

**Affiliations:** 1 XLIM, UMR-CNRS 7252, Université de Limoges, France; 2 PhLAM, UMR-CNRS 8523, Université Lille I, Villeneuve d’Ascq, France; 3 CRIBL, UMR-CNRS 7276, Université de Limoges, France; 4 Université Pierre et Marie Curie-Paris 06, LJP, F-75005 Paris, France; 5 CNRS, UMR 8237, LJP, F-75005 Paris, France; 6 Laboratoire LITIS-QuantIF, EA 4108, Clinique Pneumologique, CHU de Rouen, France

## Abstract

We present a two-photon microendoscope capable of *in vivo* label-free deep-tissue high-resolution fast imaging through a very long optical fiber. First, an advanced light-pulse spectro-temporal shaping device optimally precompensates for linear and nonlinear distortions occurring during propagation within the endoscopic fiber. This enables the delivery of sub-40-fs duration infrared excitation pulses at the output of 5 meters of fiber. Second, the endoscopic fiber is a custom-made double-clad polarization-maintaining photonic crystal fiber specifically designed to optimize the imaging resolution and the intrinsic luminescence backward collection. Third, a miniaturized fiber-scanner of 2.2 mm outer diameter allows simultaneous second harmonic generation (SHG) and two-photon excited autofluorescence (TPEF) imaging at 8 frames per second. This microendoscope’s transverse and axial resolutions amount respectively to 0.8 μm and 12 μm, with a field-of-view as large as 450 μm. This microendoscope’s unprecedented capabilities are validated during label-free imaging, *ex vivo* on various fixed human tissue samples, and *in vivo* on an anesthetized mouse kidney demonstrating an imaging penetration depth greater than 300 μm below the surface of the organ. The results reported in this manuscript confirm that nonlinear microendoscopy can become a valuable clinical tool for real-time *in situ* assessment of pathological states.

Multiphoton microscopy (MPM) is able to provide high-resolution optical sectioning several hundreds of micrometers below the surface of tissue samples without the need for labeling and with reduced phototoxicity^1^,^2^,^3^. It has been demonstrated that it represents a valuable mean for intraoperative assessment of various pathological features that could replace or at least limit surgical biopsies and related time-consuming histopathological processing^4^,^5^,^6^. For this reason MPM is destined to have a strong impact on the human clinical diagnosis in the near future^7^,^8^. The more promising configuration is to apply MPM *in vivo* deeply inside the body using a miniaturized multiphoton microscope located at the distal end of a long and flexible optical fiber. However translating MPM to clinical endoscopy and laparoscopy involves many technological challenges^9^. First of all, it has proved very difficult to deliver energetic infrared (IR) ultrashort light pulses essential for efficient multiphoton excitation at the output of a several-meter-long optical fiber, which is mandatory in a clinical environment. High resolution microendoscopy with a compact imaging probe furthermore implies the use of fiber with a small core (of a few micrometers in diameter). This dramatically reinforces the waveguide optical nonlinearity which is the cause of excitation pulse distortions and, as a consequence, increases the difficulty of pulse delivery. Another important issue is the backward collection of the visible (VIS) signals emitted by the biological sample. This is particularly true when label-free and deep imaging is sought. Indeed endogenous signals, such as cellular or tissular TPEF and SHG from extracellular matrix (ECM) type I collagen, are intrinsically quite low. Additionally, when applied to a living organism which is subjected to breath and heart movements, a two-photon microendoscope (TPME) must provide a high frame rate. It is also essential that the TPME distal tip is small enough (*i.e.* not significantly larger than 2 mm in diameter) to be introduced in the working channel of a clinical endoscope in order to allow the monitoring of the location of the microscopy probe through its wide-field imaging channel. Finally, other important characteristics of the ideal TPME rely on its field of view (FOV) and working distance (WD).

In the recent years, the above mentioned features have been the subjects of intense research efforts^10^,^11^,^12^. The preceding endeavors have mainly concerned the miniature imaging components that are embedded inside the TPME imaging probe such as fiber-scanners^13^,^14^ and micro-optics^11^,^15^,^16^,^17^. Meanwhile, the optimization of excitation IR pulse fiber delivery and the adjustment of the fiber properties, in order to optimize nonlinear VIS signal production and backward collection, have been the subject of less extensive studies. The results obtained so far were still sub-optimal thus limiting the use of TPMEs to research laboratories.

In this paper, we present an advanced TPME involving a custom-made air-silica double-clad photonic crystal fiber (DC-PCF), which is associated to an efficient fiber dispersion and nonlinearity pre-compensation device. This strategy enables the delivery of energetic sub-40 fs IR pulses at the output of a 5-m-long DC-PCF with a 3.5 μm diameter inner core. The fiber tip is inserted inside a miniature resonant fiber-scanner associated to a low demagnification achromatic compound micro-optics. Efficient backward VIS collection and guidance is obtained by means of a large area high numerical aperture concentric cladding which acts as a second collecting core around the photonic crystal microstructure. These key features allow the demonstration of a fully operating TPME system whose distal part is embedded inside a 2.2 mm outer diameter probe. In this paper, we report intrinsic TPEF and SHG fast imaging applied to a living organism. We demonstrate that our miniature fiber-based system has performances approaching those of its table-top counterparts while involving a very long and flexible optical fiber, therefore successfully meeting most of the requirements for future clinical imaging.

## Results and Discussion

### Linear and Nonlinear Pulse Shaping

The delivery of femtosecond intense light pulses through a several-meter-long optical fiber is a tricky operation, especially when pulse post-compression in free-space at the fiber end is impossible, as it is the case in microendoscopy. First, optical fibers usually have a large normal chromatic dispersion in the 700–900 nm optical window, where two-photon absorption bands of most biological intrinsic species take place^18^. As a consequence, the pulse duration strongly increases resulting in a highly reduced peak power at the fiber output. Additionally, the Kerr-induced nonlinear self-phase modulation (SPM) causes the spectral broadening of the excitation signal^19^. This nonlinear distortion is particularly exacerbated when using a small core fiber, which is mandatory for high spatial resolution fiber-optic imaging. Because of these linear and nonlinear effects, the delivered pulses are temporally and spectrally modified. As a result, the two-photon excitation at the distal end of the fiber is strongly weakened. Fiber dispersion precompensation is usually achieved by introducing an anomalous pulse stretcher before the fiber. This stretcher must be adjusted in such a way that the pulses are temporally recompressed at the fiber output. However, during propagation in the endoscopic fiber, SPM results in an unusual spectral narrowing because, in this case, it applies to negatively stretched pulses^20^. As a consequence, even with perfect temporal compression at the fiber exit, the output pulses are much longer than the initial ones. Whatever the stretcher, either a conventional grating-based stretcher^11^,^16^,^21^, a hybrid lens-grating-based stretcher^14^,^22^ or a fiber-based stretcher^23^, the distal two-photon excitation is far from being optimal. It is thus also required to compensate for the deleterious nonlinear effects occurring within the endoscopic fiber.

**Figure 1:**
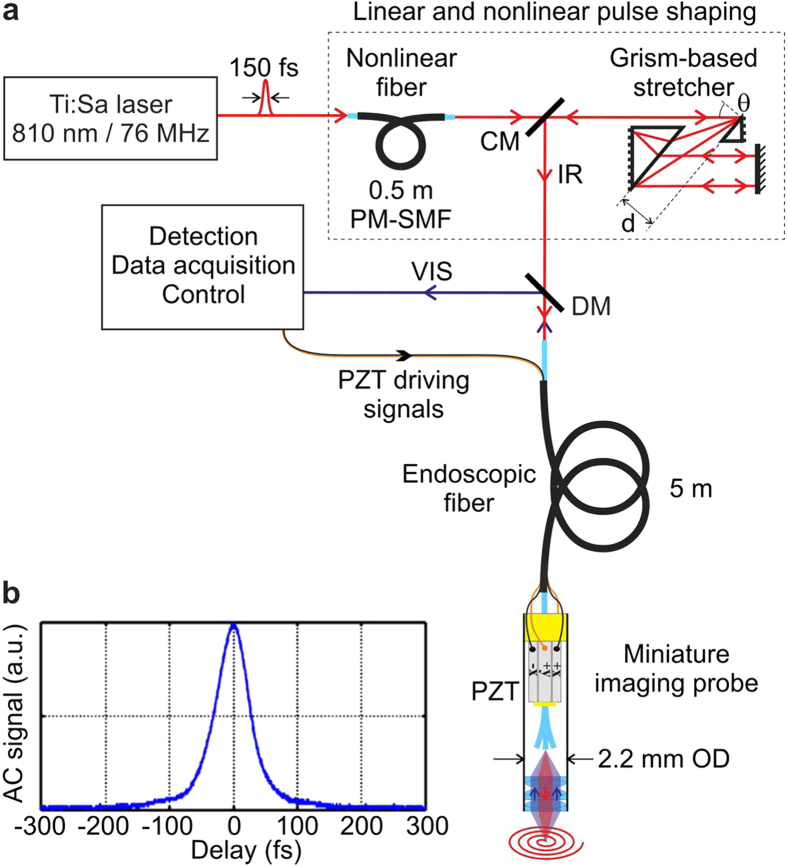
Scheme of the TPME system with linear and nonlinear pulse shaping. (**a**) Scheme of the experimetal setup; CM: cut mirror; DM: dichroic mirror; PZT: piezzoelectric tube. The miniature fiber-scanning imaging probe is embeded inside a 2.2 mm outer diameter (OD) stainless steel biocompatible tube (for more details see Supplemenatry Fig. S2a–d). (**b**) Second order autocorrelation (AC) of the IR excitation pulse at the exit of the 5-m-long endoscopic fiber for a delivered power of 20 mW. The pulse duration has been calculated from the AC duration by using the suitable conversion factor (i.e. 1.54 = (AC duration)/(pulse duration) at FWHM, assuming a sech^2^ intensity shape pulse). Accordingly, the pulse duration was equal to 39 fs (FWHM).

In the present work, the spurious nonlinear effect of spectral compression is precompensated by simply introducing a piece of standard polarization maintaining single-mode fiber (PM-SMF) acting as a nonlinear element located at the input of the system before the stretcher^24^. In this additional fiber, SPM largely broadens the spectrum because it acts upon the unchirped pulses coming directly from the femtosecond oscillator. The stretcher which is a purely linear element does not modify the spectral bandwidth of the pulses so that the spectrum at the second fiber input is very broad. This is suitable for precompensating spectral narrowing that inevitably arises in the last part of the second fiber. Moreover, this scheme allows a net reduction of the pulse duration at the system output in comparison to the input. This valuable result is achieved when spectral broadening in the first fiber is larger than spectral narrowing in the second one and when dispersion compensation is perfect. Indeed, in the middle of the system (*i.e.* inside the stretcher) the spectrum is very wide, reaching several tens of nanometers, which induces a strong sensitivity to higher-order uncompensated dispersions^24^,^25^. That is why the use of a conventional stretcher, that compensates only for second order dispersion (SOD) and brings a large amount of third order dispersion (TOD), is not optimal. We have previously demonstrated that it is possible to efficiently deliver nearly Fourier-transform limited ultrashort pulses by using a special stretcher performing simultaneous SOD and TOD compensations^26^,^27^. This stretcher involves a pair of antiparallel grisms where each *grism* is the assembly of a diffraction *gr*ating in close contact with a pr*ism*^28^,^29^. Our home-made grisms were assembled with cheap off-the-shelf commercial components. There are enough degrees of freedom within a grism-based stretcher (*i.e.* the distance *d* between the two antiparallel grisms and the incidence angle θ onto the stretcher; see Fig. 1a) to impose an accurate and independent control of both SOD and TOD. Finally, the system is limited by fourth order chromatic dispersion (FOD). Thanks to the SPM-compressed spectrum, FOD has a reduced impact and nearly Fourier-transform limited pulses are delivered at the second fiber output. From a practical point of view, the assembly, the footprint (*i.e.* 10 cm × 10 cm) and the adjustment of a grism-based stretcher are comparable to those of a conventional grating-based stretcher. When properly constructed and aligned, a grism-based stretcher is totally free from spatio-temporal aberrations (*i.e.* spatial chirp). It means that, even in the case of a very wide spectrum (*e.g.* with a full-width at half-maximum (FWHM) of about 70 nm in the experiment reported in the present paper), the whole spectrum can be injected inside the small core of a SMF (*i.e.* inside a core of about a few microns in diameter) with an injection efficiency greater than 50%. A more detailed presentation of the grism-based stretcher has been published elsewhere^29^.

In brief, our system (see Fig. 1a) comprises a standard femtosecond oscillator (150 fs, 76 MHz, 810 nm, 10 nm, 2 W), a 0.5-m-long nonlinear PM-SMF, a grism-based stretcher, a 5-m-long highly dispersive and highly nonlinear endoscopic fiber and a miniature fiber-scanning imaging probe that will be presented in the following sections. For an output power of 20 mW, the delivered pulses have a duration amounting to 39 fs (FWHM)(see Fig. 1b) (for further details see Supplementary Information (SI), Fig. S1). Remarkably, this is 3.8 times shorter than at the input. This experimental result demonstrates that we are now able to impose, at the focus of our TPME probe, a similar temporal confinement as the one usually encountered within a conventional bench-top two-photon microscope.

### Custom-designed double-clad air-silica microstructured endoscopic fiber

Hollow-core photonic crystal fibers (HC-PCF) have proved to be a valuable solution for ultrashort IR pulse delivery^30^ because it potentially enables the cancellation of fiber dispersion and nonlinearity, which would make the above mentioned advanced pre-compensation scheme useless. However this kind of innovative fiber, which has a very large fraction of air inside its microstructure, is not able to efficiently epi-collect the useful VIS signals emanating from the biological species. It has to be coupled to an additional off-axis fibered collection pathway^31^,^32^,^33^ or it can be manufactured with an additional surrounding second cladding^34^ but the related backward collection efficiency is far from being optimal. Moreover, the core diameter of a HC-PCF is quite big (largely exceeding 10 μm) which lowers the imaging resolution unless enlarging the size and the demagnification of the distal imaging optics. For these reasons, it appears that HC-PCFs are not compatible with a strong miniaturization of the TPME probe. The use of a double-clad (DC) fiber, with a small solid core for IR excitation delivery surrounded by a second large area core for VIS epi-collection, represents the most elegant solution for miniaturization^11^,^12^. Nevertheless, commercially available DCFs to date are still sub-optimal for two-photon endoscopy. Indeed, germanium-doped silica DCFs are subjected to autofluorescence around 425 nm^16^,^35^,^36^,^37^, which is detrimental when addressing weak signals emitted from intrinsic cellular and tissular autofluorescent species. A pure-silica solid-core DC-PCF, exempt from autofluorescence, has already been used within a TPME^16^,^38^,^39^. The second cladding of this fiber has a high numerical aperture (NA) which is optimal for VIS backward collection. Additionally, its large mode area (LMA) inner core has a weak optical nonlinearity that facilitates ultrashort pulse delivery. However, because of this large inner core (16 μm in diameter), the use of this kind of LMA fiber induces a detrimental loss in spatial resolution.

**Figure 2:**
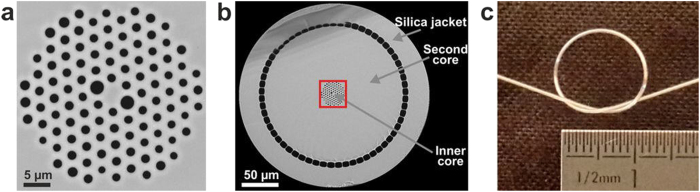
Custom-design air-silica DC-PCF used as the endoscopic fiber within the TPME. (**a**) Close view of the inner core of the fiber through scanning electron microscopy (SEM). Pure silica is in grey and air in black. (**b**) SEM image of the fiber cross-section without its outer polymer mechanical cladding. The silica jacket and the second core diameters are respectively equal to 266 μm and 188 μm. The red square denotes the inner core and its microstructured cladding which are depicted in (**a**). (**c**) DC-PCF flexibility.

In the present study, we take advantage of the powerful precompensation scheme presented in the previous section to work with a much smaller (by a factor of 4.5) pure-silica inner core DC-PCF ensuring high spatial resolution without losing the desired temporal confinement of excitation pulses. This DC-PCF was specially designed and fabricated at the PhLAM laboratory (Lille - France) using the stack and draw technique^40^. As shown in Fig. 2a,b, the DC-PCF is first composed of a small single-mode inner core of 3.5 μm diameter and 0.13 NA at 800 nm, in which IR light is guided by modified total internal reflection by a surrounding air-silica microstructured cladding (34 μm in diameter). The properties of the photonic crystal cladding ensure that the inner core is single-mode above 700 nm, which avoids intermodal dispersion that could deteriorate femtosecond pulse delivery. The chromatic dispersion is normal with SOD and TOD coefficients amounting respectively to + 228 fs^2^/cm and + 354 fs^3^/cm, representing respectively 0.66 and 1.25 times the ones of fused silica at 800 nm. The inner core, being made of pure silica, is free from autofluorescence. Moreover, two larger air holes were inserted around the core in the first ring of the microstructure (see Fig. 2a), which creates a strong group birefringence measured at 1.45 × 10^−4^ at 800 nm. As a consequence, the core is polarization maintaining with an extinction ratio higher than 15 dB, meaning that it is possible to deliver two well controlled and orthogonal linear polarization states at the fiber output. Two advantages can be drawn from this. First, the performances of the above mentioned scheme for femtosecond pulse fiber delivery are not deteriorated by bending-induced polarization mode dispersion. Additionally, the availability of two different polarization states at the fiber output will allow for endoscopic nonlinear polarimetry of biological species such as type I collagen^21^,^41^.

The inner core and its microstructured cladding are surrounded by a pure-silica second cladding acting as a second very large area collecting core of 188 μm diameter (see Fig. 2b). In this region, VIS light guidance (strongly multimode) is allowed by an outer ring of air holes, usually called “air-clad” in the literature. Its NA, measured at 0.3 at 400 nm, strongly depends on the thickness of silica bridges between air holes^42^. The overall outer diameter of the DC-PCF is 266 μm and the fiber is coated with a 120 μm thick polymer layer. As a consequence, the fiber is flexible and can be bent to a radius lower than 1 cm without breaking (see Fig. 2c), and this without any measurable bending loss for both the inner and the outer cores.

### TPEF and SHG microendoscope development

At the endoscopic fiber output, we implemented a miniaturized imaging probe composed of a resonant fiber-scanner and a micro-optics (MO) (see Fig. 1a and in SI Fig. S2a–d). The fiber-scanner and the MO are encapsulated inside a biocompatible stainless steel tube. The assembled probe is 37 mm in length and 2.2 mm in outer diameter, allowing its insertion in the working channel of a clinical endoscope or in a laparoscope. The nonlinear intrinsic luminescences emanating from the biological tissues are epi-collected by the second outer core of the DC-PCF and go back to a first dichroic mirror used to separate excitation and collection. Remaining spurious IR photons are removed with a short-pass filter and another dichroic mirror is used to separate SHG and TPEF signals. The two signals are then detected by two large active area photomultiplier tubes (PMT) (see Supplementary Text in SI for further details). They are amplified, digitalized and then sent to the computer where they are processed to form a bimodal image.

The working principle of the fiber-scanner has already been proposed by other groups^11^,^16^,^43^. A piezoelectric ceramic tube (PZT) goes around the fiber allowing to impose the trajectory of the last few millimeters of the fiber forming a resonating cantilever, thus creating an outgoing spiral scanning pattern in two dimensions. The distal fiber tip is attached to the PZT cylindrical actuator (1.5 mm of outer diameter, Physik Instrumente) through a small washer made of machinable ceramic (see yellow part in Fig. 1a and in SI Fig. S2d) with a minimal amount of glue allowing for a very high mechanical quality factor. The fiber cantilever is actuated precisely at its resonance frequency, resulting in a large maximal fiber tip deflection amounting to more than 1 mm for 60 V_pp_ onto the PZT. In order to create a well-controlled outgoing trajectory with one hundred and twenty five spirals, we specifically adjusted the electric commands sent to the four electrodes of the PZT. It is worth noticing that this kind of miniature mechanical system is inevitably anisotropic. The physical electrodes (x and y) that are implemented on the PZT do not correspond to the mechanical resonator eigen-axes (X and Y). These eigen-axes must be carefully determined during a preliminary procedure^43^. Then we also have to pre-compensate for the differential phase-shift originating from the slight difference existing between the two resonance frequencies of the two eigen-axes (see in SI Fig. S2b). The fiber tip resonates around 1416.5 Hz, with a Q-factor equal to 240 meaning that the system is very weakly damped. This high Q-factor induces transient regimes in the fiber tip movement at the beginning of the outgoing spiral, as well as during its return to its steady state onto the axis. Additional real-time numerical post-correction of the image was implemented to remove any remaining distortion induced by this transient regime. The free return of the fiber to its steady state lasts several hundreds of milliseconds. This was problematic when operating at frame rates exceeding 2 fps. This is why the return to the axis was shortened by adding a braking command provided by the PZT itself. We succeeded in shortening this braking time lapse to a duration of 12 ms (see in SI Fig. S2c) which allows for high frame rates (i.e. 8 fps). Precise control of this resonating fiber-scanner allows working with a FOV of 250 μm and 450 μm for respectively 30 V and 60 V onto the PZT, at respectively 8 fps and 4 fps, without image distortion. For a FOV of 250 μm, containing 62500 pixels, recorded at 8 fps, the pixel dwell time is equal to 1.4 μs.

Regarding the choice of a MO for the TPME, grin lenses allow for high NA and high resolution but suffer from large off-axis and chromatic aberrations while presenting a rather small WD smaller than 200 μm in water. We have decided to work with a home-built achromatic triplet considering that this solution offers a reasonable compromise between resolution (*i.e*. 0.8 μm), FOV (*i.e.* > 450 × 450 μm^2^), WD (*i.e.* 660 μm in water) and collection efficiency^16^,^44^. This triplet is the assembly of three miniature doublet achromatic lenses touching one another (see SI, at the bottom of Fig. S2d) and having a 2 mm diameter (Edmund Optics, NT65-569, *f* = 6 mm; NT65-568, *f* = 4 mm; NT65-567, *f* = 3 mm). These miniature doublets were anti-reflection coated for the 400–700 nm window. This custom-made compound system presents a NA equal to 0.45 on the sample side and 0.19 on the fiber side. The MO demagnification amounts to only 2.38 which enables a large FOV with moderate PZT drive signals. Additionally, the MO has a large WD equal to 660 μm in water.

**Figure 3:**
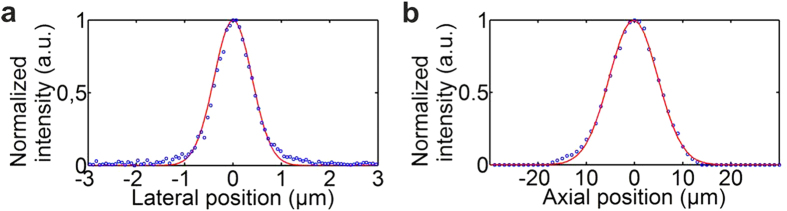
TPME optical resolutions. Intensity spatial distributions obtained during imaging a 0.1 μm-diameter fluorescent bead. Blue circle: measurements; red line: Gaussian fit. Transverse and axial resolutions were deduced from the FWHM of the Gaussian fits of the intensity distributions. (**a**) Transverse resolution: Δx = 0.83 μm. (**b**) Axial resolution: Δz = 12 μm.

The TPME transverse and axial optical resolutions were measured by imaging 0.1 μm fluorescent beads. They amount respectively to 0.83 μm and 12 μm (FWHM) (see Fig. 3). For a FOV of 250 μm in diameter, the number of resolution elements (i.e. the FOV surface divided by the point spread function surface at FWHM) is greater than 90 000.

### Biomedical validation upon *ex vivo* samples

**Figure 4 f4:**
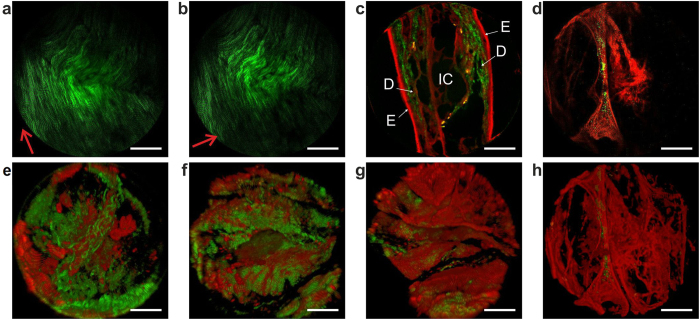
Label-free microendoscopy images of fixed tissue samples *ex vivo*. Intrinsic TPEF in red and SHG in green. (**a**–**d**) raw optical sections. (**e**–**h**) perspective view from ImageJ 3D software from a set of sixty optical sections each one corresponding to a given depth below the tissue surface, from depth 0 μm to 300 μm. (**a**,**b**) unstained intact and fresh rat tail tendon with 5 mW onto the sample. The red arrows indicate the rectilinear polarization impinging the sample. (**c**) mouse ear section. D: dermis; E: epidermis; IC: internal cartilage. (**d**): healthy human distal lung (alveolar area); alveolar wall and alveolar entrances; this optical section has been taken 100 μm below the sample surface. (**e**) perspective view of a rich collagen mouse aorta sample. (**f**–**h**): three perspective views of the extracellular matrix network at 3 different locations within a healthy human distal lung sample. (**d**,**h**) correspond to the same location. Scale bars, 50 μm.

The TPME was tested *ex vivo* on various unstained biological tissue samples. Figure 4a,b display SHG images of the collagen fiber network of a fresh rat tail tendon. It was obtained without averaging at 8 fps with only 5 mW power onto the sample proving that the TPME sensitivity is quite high. These images were obtained for two different orthogonal linear polarizations that were selected by adjusting a half-wave-plate located at the input of the PM-DC-PCF (see in SI Fig. S2a). As expected, the SHG signal was stronger when the excitation polarization was parallel to the collagen fibers^45^. This result demonstrates the ability of our TPME to perform nonlinear polarization anisotropy probing. Figure 4c displays a bimodal image of a label-free fixed section of a mouse ear. The primary constituents of the ear, the dermis, the epidermis and the internal cartilage, are clearly identified. Figure 4d shows an image recorded below the surface at a depth of 100 μm, within a human distal lung fixed sample, taken in the alveolar area. The ECM elastin fibers appear in red through intrinsic TPEF while some amount of SHG from collagen, in green, is detected entangled in the main elastin fiber. In the left part of the figure, pulmonary alveolar duct and alveolar entrances surrounded by elastin fibers can be clearly visualized. Figure 4e to h were obtained by stacking a set of sixty optical sections, each section corresponding to a given depth below the tissue surface, from 0 to 300 μm. These 3D stacks are displayed in perspective using ImageJ software. Figure 4e is a collagen rich fixed tissue of a mouse aorta while the Fig. 4f–h are distal human lung tissues. Figure 4d[Fig f4]h and the video of the successive depth-resolved optical sections that is provided in SI (see Video S1) were taken at the same location on the lung tissue sample.

### *In vivo* experiment

**Figure 5:**
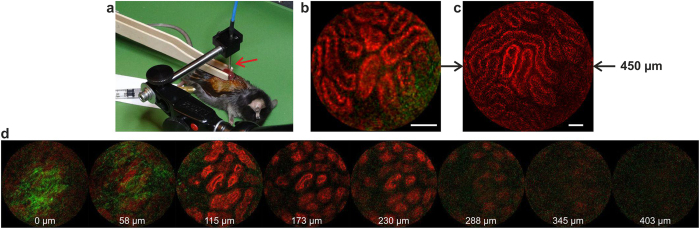
Label-free *in vivo* experiment. (**a**) Anesthetized mouse with one kidney being elevated from the body and clamped between two tongue depressors, beneath the 2.2 mm TPME probe (red arrow). A constant power of 30 mW was launched onto the tissues. (**b**) SHG (in green) and TPEF (in red) raw image of respectively the collagen of the capsule and the intracellular flavins of epithelial cells of the kidney tubules. (**c**) same as in (**b**) but with a larger FOV of 450 μm. Scale bars, 50 μm. (**d**): Successive optical sections of a fibrotic kidney, 6 days after fibrosis induction, taken just after mouse death; the imaging depth below the organ surface is indicated in the bottom in white; in (**d**), FOVs are 250 μm wide.

To the best of our knowledge only one group has published results about label-free *in vivo* two-photon microendoscopy^12^,^31^. In these studies the kidney of a healthy rat was observed. We followed the same operating method as Brown *et al.*^12^, applying it to the kidney of an anesthetized mouse (Fig. 5a). The organ was mechanically held away from the body using two tongue depressors (see Fig. 5a) to reduce motion artefacts. The structures of the kidney from its outer lining to its inner ones are first the capsule, a thin (*i.e.* a few micron-thick) layer of type I collagen, and then the kidney cells , organized in an epithelium that form tubules. Under two-photon excitation at 810 nm, the capsule can potentially be probed with SHG, while the tubules can be imaged through TPEF of intracellular flavins. Thanks to the unprecedented sensitivity of our device, we were able to clearly see the tubules and also, which is remarkable, the capsule *in vivo* in real-time at 8 fps (see Fig. 5b–d). The real-time videos of the experiment that are provided in SI (see Video S2 and Video S3) demonstrate that the TPME is not subject to motion artifact coming from animal respiration and heartbeat. A significant signal from the tubules was detected up to 300 μm below the kidney outer surface (see Fig. 5d).

**Figure 6:**
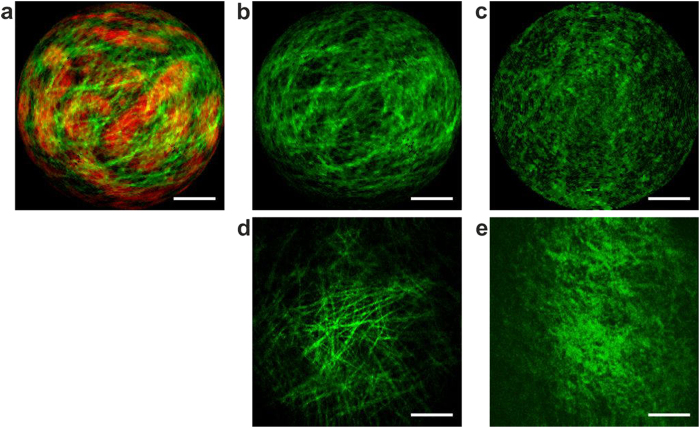
Comparison between healthy and fibrotic kidney capsules. Top line: *in vivo* label-free raw images delivered by the TPME; SHG in green and TPEF in red. (**a**,**b**) correspond to the same image, without TPEF in (**b**). Bottom line: reference *ex vivo* SHG images of the same tissues taken post-mortem with a high NA bench-top SHG microscope. (**a**,**b**,**d**) pathologic kidney, 6 days after fibrosis induction; (**c**,**e**): healthy kidney. Scale bars, 50 μm.

To further confirm the potential of our TPME, we used it to distinguish *in vivo* a healthy mouse kidney from a fibrotic one. Fibrosis was induced surgically by unilateral ureteral obstruction (UUO). Proliferation between tubules of interstitial fibroblasts with myofibroblast transformation leads to an excess deposition of the ECM, *i.e.* type I collagen, and thus can be probed with SHG^46^. The presence of inhomogeneous interstitial pathologic collagen at 6 and 13 days post UUO was confirmed on *ex vivo* kidney sections using histological dyes and also through bench-top label-free two-photon microscopy. TPME imaging sessions were performed *in vivo* on kidneys 6 and 13 days after UUO induction. Even in the case of fibrotic kidneys 13 days since the UUO, we failed to observe interstitial collagen in excess between the tubules. This can be explained considering that the fibrosis induced by UUO is spreading from the center of the kidney, while the sub-capsular areas that are accessible with the TPME are weakly concerned by interstitial collagen growth. Additionally it has been proved that this kind of collagen is a quite difficult target because of its low concentration and its inhomogeneity^46^. However looking at the capsule surface, we have clearly identified obvious differences between fibrotic kidneys (see Fig. 6b) and healthy kidneys (see Fig. 6c), even only 6 days after the UUO. The collagen fibers and the scales of the structures are larger in the diseased specimen. We confirmed this finding using a home-made high NA bench-top two-photon microscope (Fig. 6d,e). Interestingly, this result is reminiscent of those of He *et al.*^47^ and of Zhuo *et al.*^48^ who observed similar alterations of the capsule’s structure of a fibrotic liver during bench-top SHG microscopy. In addition, performing depth-resolved optical sectioning with our TPME just after the mouse’s death, we were able to detect a large increase in the thickness of the renal capsule of a post-UUO kidney. As can be seen in Fig. 5d, 58 μm below the outer surface of the kidney, the capsule is still visible in the case of a fibrotic kidney while it is not from a healthy one whose capsule is approximately 5 μm thick. This novel finding was also confirmed by observing the same kidneys *ex vivo* with a bench-top SHG microscope.

### Conclusion

We reported a multimodal TPME fulfilling most of the requirements of clinical endoscopy (*i.e.* flexibility, miniaturization), while presenting performances (sensitivity, contrast, FOV, resolution, imaging penetration depth) comparable to those of a bench-top two-photon microscope. This was achieved using a miniature 2.2-mm-diameter fiber-scanning imaging probe mounted at the output of a 5 m-long custom PM-DC-PCF. The setup presented here not only succeeds in overcoming the limitations that have previously restricted TPME fiber length to a few tens of centimeters but also produces much shorter excitation light pulses with a duration below 40 fs. This enables catching weak intrinsic signals deeply inside the diffusing biological matter with only a few milliwatts of light average power impinging the tissues. The system is capable of acquiring high content bimodal (intrinsic TPEF and SHG) images at high frame rates with a FOV of about half a millimeter deeply inside living tissues. The TPME imaging capabilities were demonstrated by imaging an *in vivo* mouse kidney through the collection of fundamental intrinsic signals without the need for staining. The results presented here open the route to minimally invasive real-time *in vivo* optical biopsies for medical clinical diagnosis. Depth-resolved imaging inside the tissues is one of the main advantages brought by multiphoton imaging (see Fig. 5d and in SI Video S1). Z-scanning was performed here using a motorized translation stage which may not be possible in clinical endoscopy. Future work about the integration of z-scanning within the miniature imaging probe, through fiber-scanning or objective-scanning^49^,^50^ or both, may definitely prove the TPME potential toward clinical imaging.

## Materials and Methods

### TPME system

The system is fed by a standard MIRA 900 Ti:Sapphire oscillator followed by a Faraday isolator avoiding reinjection back to the oscillator. More than 400 mW of the laser power are injected in the first 0.5 m-long PM-SMF in which pulses are temporally and spectrally broadened. Three different half-wave plates allow controlling the polarization where it is necessary in the setup (see in SI Fig. S2a).

### Human Tissue and Animal Preparation

The reported investigation was in accordance with the relevant guidelines for both humans and animals. The rat tail tendon was fresh while all other reported *ex vivo* samples were fixed in 4% paraformaldehyde and stored in phosphate buffered saline. The healthy human pulmonary tissue samples, prepared at Rouen University Hospital, were obtained from a lobectomy. They were sampled from a healthy area of a pulmonary lobe. According to the French regulation, the use of the human tissue sample for the experiment was approved by scientific committee of the Rouen University Hospital Tumor Tissue Bank, and a written informed consent obtained from the patient. Induction of UUO in mice and the preparation of animals for *in vivo* imaging sessions were carried out by the staff of the UMR CNRS 7276 in accordance with European regulations, applied in France by Decret No. 2013-118 of 1st February 2013 on the protection of animals used by scientists. The protocol was approved by the ethics committee for animals used by scientists registered under the code C2A2-33 by the French “Ministère de l’Education Nationale, de l’Enseignement Supérieur et de la Recherche”. During *in vivo* imaging sessions, the preparation and observation of the mice were made with a protocol similar to the one described by Brown *et al.*^12^, the main difference being the anesthesia. In our case, mice received a non-barbiturate anesthetic and a muscle relaxant sedative analgesic (ketamine at 18 mg/mL and xylazine at 0.9 mg/mL) through intraperitoneal injection. The kidney, elevated from the body, was clamped between two tongue depressors and placed on the flank of the mice, beneath the probe (see Fig. 5a). The TPME probe was manipulated by means of a 3-axis precision motorized micromanipulator (Thorlabs, Inc., MAX 343). The micromanipulator was connected to an articulated arm (see Fig. 5a) holding the TPME distal tip. The depth resolved imaging results reported in Fig. 4e–h and in Fig. 5d were obtained with this micromanipulator that allows precisely controlling the z coordinate.

## Additional Information

**How to cite this article**: Ducourthial, G. *et al.* Development of a real-time flexible multiphoton microendoscope for label-free imaging in a live animal. *Sci. Rep.* **5**, 18303; doi: 10.1038/srep18303 (2015).

## Supplementary Material

Supplementary Information

Supplementary Video S1

Supplementary Video S2

Supplementary Video S3

## References

[b1] Denk W., Strickler J. & Webb W. Two-photon laser scanning fluorescence microscopy. Science 248, 73–76 (1990). 2321027 10.1126/science.2321027

[b2] Masters B. R. & So P. T. Biomedical applications of nonlinear optical microscopy in Handbook of biomedical nonlinear optical microscopy 705–844 (Oxford University, 2008).

[b3] Hoover E. E. & Squier J. A. Advances in multiphoton microscopy technology. Nat. Photonics 7, 93–101 (2013). 24307915 10.1038/nphoton.2012.361 PMC3846297

[b4] Skala M. C. *et al.* Multiphoton microscopy of endogenous fluorescence differentiates normal, precancerous, and cancerous squamous epithelial tissues. Cancer Res. 65, 1180–1186 (2005). 15735001 10.1158/0008-5472.CAN-04-3031 PMC4189807

[b5] Wang C.-C. *et al.* Differentiation of normal and cancerous lung tissues by multiphoton imaging. J. Biomed. Opt. 14, 044034 (2009). 19725745 10.1117/1.3210768

[b6] Tao Y. K. *et al.* Assessment of breast pathologies using nonlinear microscopy. Proc. Natl. Acad. Sci. 111, 15304–15309 (2014). 25313045 10.1073/pnas.1416955111 PMC4217415

[b7] Jain M. *et al.* Multiphoton microscopy: a potential ‘optical biopsy’ tool for real-time evaluation of lung tumors without the need for exogenous contrast agents. Arch. Pathol. Lab. Med. 138, 1037–1047 (2014). 24199831 10.5858/arpa.2013-0122-OA

[b8] Perry S. W., Burke R. M. & Brown E. B. Two-Photon and Second Harmonic Microscopy in Clinical and Translational Cancer Research. Ann. Biomed. Eng. 40, 277–291 (2012). 22258888 10.1007/s10439-012-0512-9 PMC3342697

[b9] Flusberg B. A. *et al.* Fiber-optic fluorescence imaging. Nat Meth 2, 941–950 (2005). 10.1038/nmeth820 PMC2849801 16299479

[b10] Bao H., Allen J., Pattie R., Vance R. & Gu M. Fast handheld two-photon fluorescence microendoscope with a 475 μm × 475 μm field of view for *in vivo* imaging. Opt. Lett. 33, 1333–1335 (2008). 18552949 10.1364/ol.33.001333

[b11] Zhang Y. *et al.* A compact fiber-optic SHG scanning endomicroscope and its application to visualize cervical remodeling during pregnancy. Proc. Natl. Acad. Sci. 109, 12878–12883 (2012). 22826263 10.1073/pnas.1121495109 PMC3420182

[b12] Brown C. M. *et al.* *In vivo* imaging of unstained tissues using a compact and flexible multiphoton microendoscope. J. Biomed. Opt. 17, 0405051–0405053 (2012). 10.1117/1.JBO.17.4.040505 PMC3382343 22559671

[b13] Myaing M. T., MacDonald D. J. & Li X. Fiber-optic scanning two-photon fluorescence endoscope. Opt. Lett. 31, 1076–1078 (2006). 16625908 10.1364/ol.31.001076

[b14] Rivera D. R. *et al.* Compact and flexible raster scanning multiphoton endoscope capable of imaging unstained tissue. Proc. Natl. Acad. Sci. 108, 17598–17603 (2011). 22006303 10.1073/pnas.1114746108 PMC3203813

[b15] Wu Y., Xi J., Cobb M. J. & Li X. Scanning fiber-optic nonlinear endomicroscopy with miniature aspherical compound lens and multimode fiber collector. Opt. Lett. 34, 953–955 (2009). 19340182 10.1364/ol.34.000953 PMC2697571

[b16] Zhao Y., Nakamura H. & Gordon R. J. Development of a versatile two-photon endoscope for biological imaging. Biomed. Opt. Express 1, 1159–1172 (2010). 21258538 10.1364/BOE.1.001159 PMC3018080

[b17] Ouzounov D. G., Rivera D. R., Webb W. W., Bentley J. & Xu C. Miniature varifocal objective lens for endomicroscopy. Opt. Lett. 38, 3103–3106 (2013). 24104660 10.1364/OL.38.003103

[b18] Zipfel W. R. *et al.* Live tissue intrinsic emission microscopy using multiphoton-excited native fluorescence and second harmonic generation. Proc. Natl. Acad. Sci. 100, 7075–7080 (2003). 12756303 10.1073/pnas.0832308100 PMC165832

[b19] Agrawal G. Self-phase modulation in Nonlinear Fiber Optics 79–116 (A. Press, 2007).

[b20] Oberthaler M. & Höpfel R. Special narrowing of ultrashort laser pulses by self‐phase modulation in optical fibers. Appl. Phys. Lett. 63, 1017–1019 (1993).

[b21] Bao H., Boussioutas A., Jeremy R., Russell S. & Gu M. Second harmonic generation imaging via nonlinear endomicroscopy. Opt. Express 18, 1255–1260 (2010). 20173949 10.1364/OE.18.001255

[b22] Durst M. E., Kobat D. & Xu C. Tunable dispersion compensation by a rotating cylindrical lens. Opt. Lett. 34, 1195–1197 (2009). 19370115 10.1364/ol.34.001195

[b23] Wu Y., Leng Y., Xi J. & Li X. Scanning all-fiber-optic endomicroscopy systemfor 3D nonlinear optical imaging of biologicaltissues. Opt. Express 17, 7907–7915 (2009). 19434122 10.1364/oe.17.007907 PMC2696815

[b24] Clark S. W., Ilday F. O. & Wise F. W. Fiber delivery of femtosecond pulses from a Ti:sapphire laser. Opt. Lett. 26, 1320–1322 (2001). 18049595 10.1364/ol.26.001320

[b25] Lelek M. *et al.* Coherent femtosecond pulse shaping for the optimization of a non-linear micro-endoscope. Opt. Express 15, 10154–10162 (2007). 19547364 10.1364/oe.15.010154

[b26] Lefort C., Mansuryan T., Louradour F. & Barthelemy A. Pulse compression and fiber delivery of 45 fs Fourier transform limited pulses at 830 nm. Opt. Lett. 36, 292–294 (2011). 21263530 10.1364/OL.36.000292

[b27] Lefort C. *et al.* Sub-30-fs pulse compression and pulse shaping at the output of a 2-m-long optical fiber in the near-infrared range. J. Opt. Soc. Am. B 31, 2317–2324 (2014).

[b28] Tournois P. New diffraction grating pair with very linear dispersion for laser pulse compression. Electron. Lett. 29, 1414–1415 (1993).

[b29] Kalashyan M. *et al.* Ultrashort pulse fiber delivery with optimized dispersion control by reflection grisms at 800 nm. Opt. Express 20, 25624–25635 (2012). 23187381 10.1364/OE.20.025624

[b30] Skibina J. S. *et al.* A chirped photonic-crystal fibre. Nat Photon 2, 679–683 (2008).

[b31] Ouzounov D. G. *et al.* Dual modality endomicroscope with optical zoom capability. Biomed. Opt. Express 4, 1494–1503 (2013). 24049671 10.1364/BOE.4.001494 PMC3771821

[b32] Le Harzic R., Weinigel M., Riemann I., König K. & Messerschmidt B. Nonlinear optical endoscope based on a compact two axes piezo scanner and a miniature objective lens. Opt. Express 16, 20588–20596 (2008). 19065197 10.1364/oe.16.020588

[b33] Engelbrecht C. J., Johnston R. S., Seibel E. J. & Helmchen F. Ultra-compact fiber-optic two-photon microscope for functional fluorescence imaging *in vivo*. Opt. Express 16, 5556–5564 (2008). 18542658 10.1364/oe.16.005556

[b34] Brustlein S. *et al.* Double-clad hollow core photonic crystal fiber for coherent Raman endoscope. Opt. Express 19, 12562–12568 (2011). 21716497 10.1364/OE.19.012562

[b35] Udovich J. A. *et al.* Spectral background and transmission characteristics of fiber optic imaging bundles. Appl. Opt. 47, 4560–4568 (2008). 18758526 10.1364/ao.47.004560

[b36] Chang Y.-C. *et al.* Two-photon fluorescence correlation spectroscopy through a dual-clad optical fiber. Opt. Express 16, 12640–12649 (2008). 18711501 10.1364/oe.16.012640

[b37] Peyrot D. A. *et al.* Development of a nonlinear fiber-optic spectrometer for human lung tissue exploration. Biomed. Opt. Express 3, 840–853 (2012). 22567579 10.1364/BOE.3.000840 PMC3342191

[b38] Fu L., Jain A., Xie H., Cranfield C. & Gu M. Nonlinear optical endoscopy based on a double-clad photonic crystal fiber and a MEMS mirror. Opt. Express 14, 1027–1032 (2006). 19503423 10.1364/oe.14.001027

[b39] Fu L. & Gu M. Fibre-optic nonlinear optical microscopy and endoscopy. J. Microsc. 226, 195–206 (2007). 17535259 10.1111/j.1365-2818.2007.01777.x

[b40] Russell P. Photonic Crystal Fibers. Science 299, 358–362 (2003). 12532007 10.1126/science.1079280

[b41] Fu L. & Gu M. Polarization anisotropy in fiber-optic second harmonic generation microscopy. Opt. Express 16, 5000–5006 (2008). 18542600 10.1364/oe.16.005000

[b42] Wadsworth W. J. *et al.* Very high numerical aperture fibers. IEEE Photonics Technol. Lett. 16, 843–845 (2004).

[b43] Kundrat M. J., Reinhall P. G., Lee C. M. & Seibel E. J. High performance open loop control of scanning with a small cylindrical cantilever beam. J. Sound Vib. 330, 1762–1771 (2011). 21359102 10.1016/j.jsv.2010.10.019 PMC3045204

[b44] Wu Y. & Li X. Combined influences of chromatic aberration and scattering in depth-resolved two-photon fluorescence endospectroscopy. Biomed. Opt. Express 1, 1234–1243 (2010). 21258545 10.1364/BOE.1.001234 PMC3018084

[b45] Williams R. M., Zipfel W. R. & Webb W. W. Interpreting Second-Harmonic Generation Images of Collagen I Fibrils. Biophys. J. 88, 1377–1386 (2005). 15533922 10.1529/biophysj.104.047308 PMC1305140

[b46] Strupler M. *et al.* Second harmonic imaging and scoring of collagen in fibrotic tissues. Opt. Express 15, 4054–4065 (2007). 19532649 10.1364/oe.15.004054

[b47] He Y. *et al.* Toward surface quantification of liver fibrosis progression. J. Biomed. Opt. 15, 056007–056007–11 (2010). 21054101 10.1117/1.3490414

[b48] Zhuo S. *et al.* *In vivo*, label-free, three-dimensional quantitative imaging of liver surface using multi-photon microscopy. Appl. Phys. Lett. 105, 023701 (2014).

[b49] Kim M. *et al.* Miniature objective lens with variable focus for confocal endomicroscopy. Biomed. Opt. Express 5, 4350 (2014). 25574443 10.1364/BOE.5.004350 PMC4285610

[b50] Zhou G., Yu H. & Chau F. S. Microelectromechanically-driven miniature adaptive Alvarez lens. Opt. Express 21, 1226 (2013). 23389015 10.1364/OE.21.001226

