# Supplementary material for: Development of a real-time flexible multiphoton microendoscope for label-free imaging in a live animal
Source: Sci Rep. 2015 Dec 17;5:18303. doi: 10.1038/srep18303 (PMC4682136; doi:10.1038/srep18303)
Supplement: Supplementary Information [file srep18303-s1.pdf]

Development of a real-time flexible multiphoton microendoscope for label-free imaging in a live animal

Supplementary Information

Guillaume Ducourthial[1], Pierre Leclerc[1], Tigran Mansuryan[1], Marc Fabert[1], Julien Brevier[1], Rémi Habert[2], Flavie Braud[2], Renaud Batrin[3], Christine Vever-Bizet[4, 5], Geneviève Bourg-Heckly[4, 5], Luc Thiberville[6], Anne Druilhe[3], Alexandre Kudlinski[2], Frédéric Louradour[1*]

[1]XLIM, UMR-CNRS 7252, Université de Limoges, France ; [2]PhLAM, UMR-CNRS 8523, Université Lille I, Villeneuve d'Ascq, France ; [3]CRIBL, UMR-CNRS 7276, Université de Limoges, France ; [4]Université Pierre et Marie Curie-Paris 06, LJP, F-75005 Paris, France; [5]CNRS, UMR 8237, LJP, F-75005 Paris, France; [6]Laboratoire LITIS-QuantIF, EA 4108, Clinique Pneumologique, CHU de Rouen, France.

*Corresponding author: louradour@xmim.fr

Supplementary Text

Detection setup. Intrinsic 2PEF and SHG signal are epi-collected through the core and inner clad of the DC-PCF. Back to the proximal unit, the useful VIS responses are discriminated from the IR excitation by a first dichroic beam-splitter (Semrock, FF705-Di01-25x36; DM1 in Supplementary Fig. S2a) followed by a rejection filter (Semrock, FF01-720/SP-25; F1 in Supplementary Fig. S2a). SHG is extracted by a second dichroic beam-splitter (Semrock, Di02-R442-25x36; DM2 in Supplementary Fig. S2a) which is completed by a 10 nm-bandpass filter centered at 405 nm (Semrock, FF01-405/10-25; F2 in Supplementary Fig. S2a). Finally, 2PEF and SHG are detected by two different cooled PMTs (Hamamatsu, H7422-40P with a maximal quantum efficiency amounting to 36%), working in analogic detection mode or in 100 MHz photon counting, and digitized by a data acquisition card (National Instruments, PCI6115). A particular

attention has been devoted to the proper focusing onto the detectors of the useful signals exiting the high NA multimode second core of the DC-PCF. Specific matching optics was added for that (not shown).

Supplementary Figures

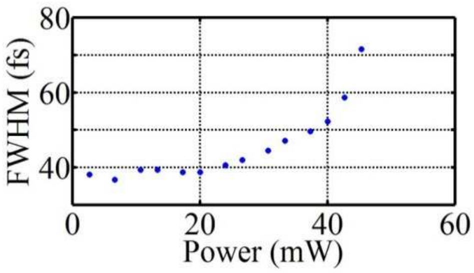

Supplementary Fig. S1: Pulse duration at the TPME output as a function of output power. Above 20 mW, the compressed pulse duration increases as a result of nonlinear spectral compression taking place in the last few millimeters of the endoscopic fiber. Even at 40 mW the final pulse duration is still 3 times shorter than at the direct exit of the laser that feeds the system.

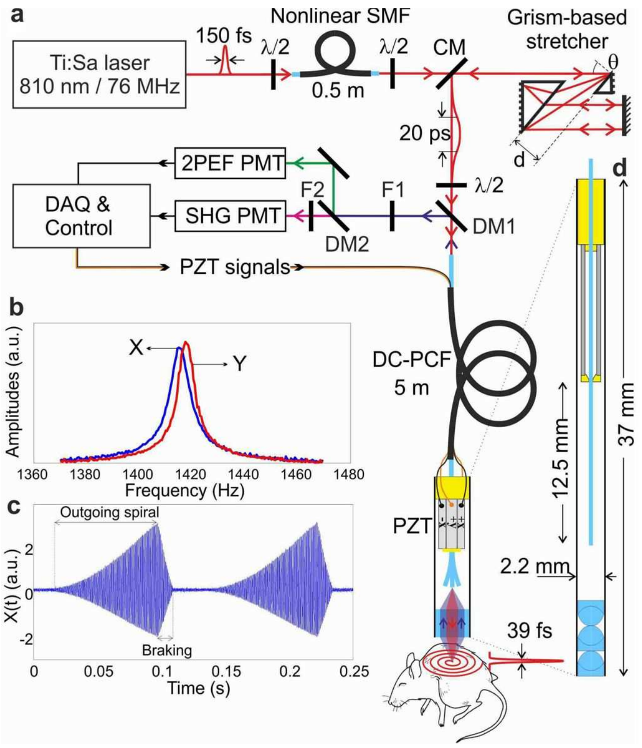

Supplementary Fig. S2: TPME system and miniature fiber-scanner. (**a**) Scheme of the experimetal setup. SMF: single-mode fiber; CM: cut mirror; λ/2: half-wave plate, DM: dichroic mirror; F: filter; PMT: photomultiplier tube; DAQ: data acquisition. (**b**) Resonance of the fiber-scanner. X and Y: eigen-axes of the mechanical resonator. (**c**) Evolution of the oscillations of the scanning fiber tip along X axis recorded with a position sentive detector (PSD Module C10443-04, Hamamatsu). At the end of the one hundred and twenty five spirals, the peak-to-peak amplitude of oscillation is larger than 1 mm for 60 V_{pp} onto the PZT. Active braking is completed in 12 ms. (**d**) Wide plan of the imaging probe with the fiber and the imaging triplet composed of achromatic doublets in sky blue, the PZT in grey and the linking pieces made of machinable ceramic in yellow. The probe is embeded inside a 2.2 mm outer diameter stainless steel biocompatible tube.

Supplementary Videos

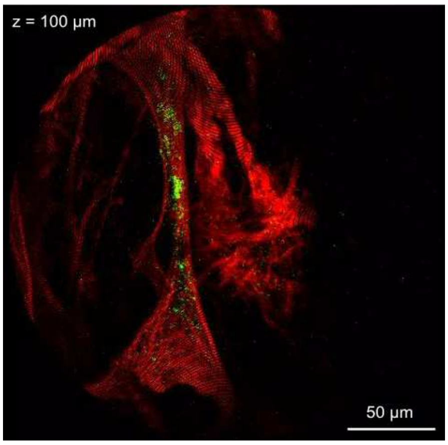

Supplementary Video S1. Label-free microendoscopy successive sections within the extracellular matrix network of a fixed healthy human distal lung tissue sample *ex vivo*. Movie of the successive optical sections during the variation of the imaging penetration depth from depth z = 0 µm to z = 300 µm. Intrinsic 2PEF in red and SHG in green.

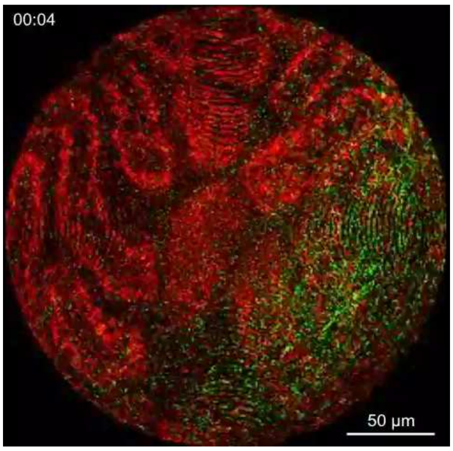

Supplementary Video S2. Real-time video at 8 fps of *in vivo* label-free experiment upon an anesthetized mouse kidney. Collagen, from SHG in green, of the kidney capsule and intracellular flavins, from intrinsic TPEF in red, of epithelial cells of the kidney tubules. The animal heartbeat and respiration does not perturb image recording. The real time of the experiment is indicated at top left. FOV, 250 µm. Thin periodic striations

can be seen on the borders of the FOV. This small artifact is due to a slight mismatch between the image sampling imposed by the spiral scanning fiber-scanner and the final image which is a rectangular matrix of pixels.

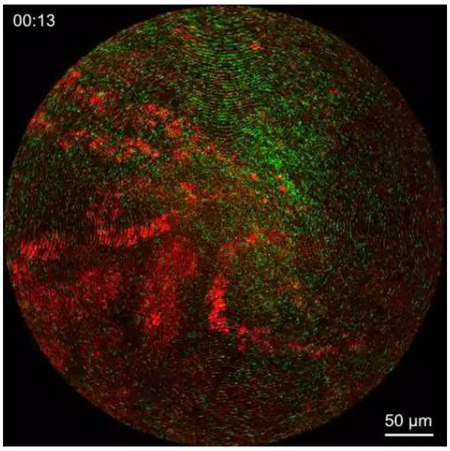

Supplementary Video S3. Same as Supplementary Video S2 but for a FOV of 450 µm and with 4 fps. The real time of the experiment is indicated at top left.
